# Older Adults’ Information Use on Social Media: The Role of Psychological Needs and Personality Traits

**DOI:** 10.1177/01640275251341447

**Published:** 2025-05-14

**Authors:** Luise Anter, Martin Fischer, Anna Sophie Kümpel

**Affiliations:** 1 Department of Media and Communication, 9183 LMU Munich, Munich, Germany

**Keywords:** information use, older adults, psychological needs, social media, survey

## Abstract

This study explores how basic psychological needs and personality traits relate to the types of information that older adults use on social media platforms. Relying on a nationally representative survey of 1100 German Facebook and Instagram users aged 60+, it examines the relationship between three psychological needs—autonomy, competence, and relatedness—and four types of information use: undirected, topic-related, group-related, and problem-related. The study also explores associations with fear of missing out (FOMO), political interest, and openness to experience. Findings indicate that the need for relatedness and FOMO are key predictors of older adults’ information use, emphasizing the social motivations behind their social media engagement. Notably, Facebook is more frequently used than Instagram across all types of information use. These insights provide a deeper understanding of the interactions between psychological needs, personality traits, and information behaviors among older adults, informing future research on their social media usage.

With the growing digitization of communication, older adults are increasingly integrating social media platforms such as Facebook and Instagram into their information repertoires (see, for example, for the USA: Kakulla, 2024; for Germany: Müller, 2024). Accordingly, researchers have started to examine older adults’ use of social media, often focusing on their overarching motives for using these platforms. Social interaction has been identified as a key driver (e.g., Bernardo et al., 2022), but older adults also use content related to health (e.g., Braun et al., 2019), travel (e.g., Darley et al., 2017), or news (e.g., Coelho, 2024). While these studies provide preliminary insights into the general adoption of social media among older adults, evidence on the specific types of information they use remains limited. Moreover, many studies rely on qualitative methods (e.g., Coelho, 2024; Zhao et al., 2023), which offer valuable in-depth insights but are often restricted by small sample sizes and lack standardized evidence.

Besides, existing research often focuses on specific topics, such as health or social interaction, which implicitly frames older adults in predefined roles, such as ‘patients’ or ‘loneliness sufferers.’ In contrast to research on younger adults’ social media information use (e.g., Bloemen & De Coninck, 2020; Gil de Zúñiga et al., 2019), studies also primarily provide anecdotal evidence regarding the factors *influencing* older adults’ social media information behavior, highlighting the need for further investigation. For instance, Zhao et al. (2023) identified a complex relationship between older adults’ basic psychological needs (Ryan & Deci, 2017) and social media use. While stronger needs can drive increased usage, they may also lead to frustration if older adults feel overwhelmed by the platforms or unable to fulfill their needs, potentially resulting in reduced engagement. Additionally, variations in social media use have been linked to factors such as political interest (e.g., Singh, 2015) and the need for social integration (e.g., Wang et al., 2018).

In light of these findings, this study aims to provide standardized and systematic evidence on the types of information older adults use on Facebook and Instagram—the two most popular platforms in this demographic (Koch, 2023)—and to examine how the strength of individuals’ basic psychological needs such as relatedness (*RQ1*) and personality traits related to information behavior, namely the fear of missing out (FOMO), political interest, and openness to experience (*RQ2*), predict this usage. To address these research questions, we adopt a social understanding of information, defining it as personally new and/or valuable (Hasebrink & Domeyer, 2010). This perspective allows us to distinguish between various types of information, such as news or content related to personal interests and hobbies.

Employing a pre-registered, nationally representative survey of German Facebook and Instagram users aged 60 and over (*n* = 1100), we find that older adults’ social media use is closely related to their need for relatedness and FOMO, highlighting the social origin of their social media engagement. Furthermore, the platform used emerged as a significant factor, suggesting that older adults may perceive Facebook as more suitable for accessing information than Instagram. Based on these findings, we discuss the unique characteristics of older adults’ information use on social media and propose directions for future research.

## Social Media Information Use by Older Adults

A significant body of literature highlights social interaction as a key motivator for older adults adopting social media (e.g., Wang et al., 2018). Older adults frequently use platforms like Facebook to stay informed about their close social circles or to “keep track of the grandkids” (Freeman et al., 2020, p. 6), as well as to maintain relationships with broader networks (e.g., Huisman et al., 2020).

However, studies also indicate that older adults value the diversity of topics and viewpoints available on these platforms (Balki et al., 2023). They use social media not only for social interactions but also to obtain health information (e.g., Chen et al., 2021) or to plan and share travel experiences (e.g., Darley et al., 2017). An in-depth study by Bansal and Choudhary (2023) further reveals that older adults engage with a variety of content, including political issues, cultural events, and physical exercise (see also Zhao et al., 2023). Moreover, there is evidence that, although often incidentally, older adults encounter news on social media (e.g., Coelho, 2024).

Building on these findings, we adopt a social definition of information (Hasebrink & Domeyer, 2010), conceptualizing it as both novel and useful/valuable from a subjective perspective. Hasebrink and Domeyer (2010) theoretically identify four distinct information needs, each leading to different types of information use (ibid.). First, people have a general desire for information about their environment (*undirected information needs*), leading them to use news or general life management information (henceforth: undirected information)*. Topic-related information needs* stem from individuals’ need to stay informed about personal interests and hobbies, resulting in the use of information about specific subject areas relevant to them (henceforth: topic-related information). To socially integrate, individuals have *group-related information needs*, which lead them to use information about their closer and wider social circles, such as family, friends, or colleagues (henceforth: group-related information). Finally, *problem-related information needs* develop as individuals seek solutions for specific problems or ways to manage particular situations. Thus, people may seek problem-related information such as product descriptions or lunch recipes.

While these information needs are universal, their relevance can vary depending on personality and other factors. For example, one older adult may be highly interested in current affairs or cooking, but less concerned with their niece’s latest travel experience. To better understand and systematically assess the differences in older adults’ information use on social media, we draw on the framework of self-determination theory, which identifies fundamental psychological needs, and consider additional personality traits that may shape information use behaviors.

### The Role of Basic Psychological Needs

Self-determination theory (SDT) is a psychological framework for exploring “the social conditions that facilitate or hinder human flourishing” (Ryan & Deci, 2017, p. 3 see also Deci & Ryan, 1985). The term self-determination refers to an individual’s ability to organize their actions, think independently, and make conscious decisions. According to SDT, in addition to physiological needs (e.g., nutrition, rest, oxygen), people have psychological needs: autonomy, competence, and relatedness. These psychological needs are fundamental drivers of human behavior, and their fulfillment leads to subjective well-being (e.g., Reis et al., 2000).

*Autonomy* refers to the ability to regulate one’s actions and experiences independently, while *competence* involves feeling effective and capable in one’s activities. For older adults, autonomy and competence encompass managing their lives without the help of others (Ferrand et al., 2014). These needs may drive undirected information use, as older adults turn to social media to navigate their environment, stay informed about local events, or access news. Additionally, competence may be linked to topic-related and problem-related information use. To feel competent and achieve a sense of accomplishment, older adults may turn to platforms like Facebook or Instagram to acquire new skills or seek information that helps solve specific problems (Zhao et al., 2023). *Relatedness* is the need to feel socially connected and integrated into social networks—a crucial factor for older adults (Custers et al., 2012). This need may drive group-related information use, such as following family members’ activities or sharing personal experiences.

SDT has been widely applied to study media use, including older adults’ engagement with mobile apps (Zhang et al., 2024) or the Internet in general (Schreurs et al., 2017). While research often examines the *satisfaction* of psychological needs as an *outcome* of media use and its relation to well-being, we conceptualize the extent to which people *possess* these needs in the first place as a *reason* for using information on social media. Although relatedness, competence, and autonomy are innate needs, individuals may prioritize them differently (Karahanna et al., 2018). Older adults with a strong need for competence may engage more with hobby-related information on social media. Similarly, those interested in learning new things are more likely to use tutorials or topic-specific groups on social media (Zhao et al., 2023) Thus, individual differences in autonomy, competence, and relatedness help explain variations in information use on platforms like Facebook and Instagram.

Overall, the literature supports the idea that the three basic psychological needs serve as intrinsic motivators for information use on social media (e.g., Sheldon & Titova, 2023; Wang & Li, 2016). For instance, Wei et al. (2021) found that individuals with a higher need for relatedness use social media more frequently for private purposes, while those with a higher need for competence use it more for work-related purposes. Similarly, individuals experiencing a lack of relatedness tend to use social media more for group-related purposes, whereas those striving for competence use it more for information seeking (e.g., Sheldon et al., 2011). Interviews conducted by Zhao et al. (2023) revealed that older adults with an interest in their environment and a desire to learn (indicating high competence and autonomy needs) engaged with diverse types of information on social media, ranging from COVID-19 news to tutorials and yoga courses. Additionally, participants who felt restricted in their social networks during the pandemic (indicating a high relatedness need) reported increased use of Facebook to connect with their local community.

In summary, these findings indicate a positive association between the extent or strength of psychological needs and various types of social media information use. However, research also shows that perceptions of social media can influence this relationship. For example, need satisfaction is hindered if individuals distrust social media (Wang & Li, 2016) or find it unenjoyable (Sheldon & Titova, 2023). Indeed, older adults frequently find social media overwhelming and complicated (Meng, 2024). As a result, having a strong psychological need may not necessarily lead older adults to use social media for specific types of information. Given the complexity of older adults’ social media use and the limited research on their information behaviors, we pose a broad question to comprehensively explore how psychological needs shape information use:

**RQ1:** How do the three basic psychological needs (autonomy, competence, and relatedness) influence the frequency of older adults’ use of different types of information on social media platforms?

### The Role of Personality Traits

While basic psychological needs offer insight into the intrinsic motivations behind older adults’ use of Facebook and Instagram, research suggests that personality traits also shape their information behavior on social media. Therefore, we also explore three key personality traits: *fear of missing out* (FOMO), *political interest*, and *openness to experience*. We deliberately focus on these well-established constructs from psychology and media research, as prior studies indicate their particular relevance to different dimensions of information use, as we will illustrate in the following sections.

*FOMO* may be an important factor influencing group-related information use. It is defined as a “pervasive apprehension that others might be having rewarding experiences from which one is absent” (Przybylski et al., 2013a, p. 1841), creating a desire to stay constantly connected to others’ activities. FOMO drives individuals to continuously seek social information to ascertain their position within social hierarchies (Przybylski et al., 2013b). Social media provides an ideal platform for maintaining connections with one’s social environment, and in adolescents, FOMO has been linked to more intensive social media use (e.g., Bloemen & De Coninck, 2020). Therefore, it is plausible that older adults with higher levels of FOMO also use social media more frequently for group-related information.

To understand undirected information use, we focus on *political interest*, defined as the “degree to which politics arouses a citizen’s curiosity” (van Deth, 1990, p. 289) and the attention individuals pay to politics. Research indicates that politically interested users consume more news, seek it more actively, and have greater political knowledge than those with lower political interest (e.g., Shehata & Strömbäck, 2021). In contrast, low political interest is associated with minimal news consumption, increased news avoidance, and the news-finds-me perception—the belief that one can stay informed without actively seeking news (Gil de Zúñiga & Diehl, 2019; e.g., Goyanes et al., 2021). These findings suggest that politically interested older adults are more likely to use undirected information on Facebook and Instagram.

Finally, *openness to experience*, one of the five major personality traits, refers to individuals’ curiosity, breadth of interests, and receptiveness to new experiences (McCrae & Greenberg, 2014). It is, for instance, associated with a broader array of personal interests (e.g., Matz, 2021). In the context of social media, individuals with higher openness to experience tend to engage with more accounts, join more groups, and show interest in a variety of events (Saef et al., 2018). Additionally, openness to experience is positively correlated with the need for cognition—the extent to which individuals engage in and enjoy complex cognitive activities (Sadowski & Cogburn, 1997). Therefore, older adults with higher openness to experience may use social media more frequently for problem-related information, finding satisfaction in the process of searching for and learning new information on these platforms.

Of course, these personality traits may relate to all four types of information use. For instance, FOMO has also been linked to personal news curation practices on social media (Wu-Ouyang, 2024), while openness to experience is associated with using social media for both socializing and gathering news (Hughes et al., 2012). Additionally, political interest is associated with greater relevance of all four information needs (Kümpel et al., 2022). Therefore, our study aims to explore the broader relevance of these personality traits across all four types of information use, ranging from undirected to problem-related information. We pose the following research question:

**RQ2:** How do additional personality traits (FOMO, political interest, and openness to experience) influence the frequency of older adults’ use of different types of information on social media platforms?

## Method

To address our research questions, we conducted a preregistered cross-sectional online survey with *n* = 1100 German social media users (≥60 years) in August/September 2024. Data, materials, and scripts to reproduce the analyses are available at https://osf.io/sd2t5/?view_only=0343cee654c14ab9bb83278bb9fe54cb, the preregistration is available at https://osf.io/6ucm2/?view_only=368a3adf53b24e69ba53e2998f47a02a.

### Participants

Participants were recruited through a commercial online access panel hosted by the established professional provider *Bilendi*. The sample was drawn based on nationally representative quotas for gender (male, female), education (low, medium, high), and region (federal state). To be eligible for participation, individuals had to reside in Germany, be 60 years of age or older, and use Facebook or Instagram at least occasionally. We selected these platforms not only because they are the most widely used among this demographic in Germany (Koch, 2023), but also due to their distinct designs. For instance, they differ in their level of visuality and network structure, with Instagram being more visually oriented and Facebook emphasizing network-based interactions (Bossetta, 2018).

Participants who did not meet the eligibility criteria, failed either of the two attention checks included in the questionnaire, or completed the survey in an unusually short time (i.e., “speeders”, see Zhang & Conrad, 2014) were excluded during the field phase as part of a quality check. A total of 2532 respondents accessed the questionnaire. Of these, 1432 were excluded due to quota constraints, not meeting our eligibility criteria and/or insufficient response quality.

Sociodemographic characteristics of the final sample of 1100 participants include gender (49.9% female), age (*M* = 67.20 years, *SD* = 5.41, range 60–88), and highest educational degree completed (17.1% held a university degree). This distribution reflects broader trends in social media use among German older adults, where men and women participate at similar rates, and individuals with lower educational attainment tend to use social media slightly more frequently than those with higher education (Rathgeb et al., 2022). Regarding social media usage, participants reported using Facebook (*M* = 5.12, *SD* = 2.21, scale ranging from *never* [0] to *several times a day* [7]) more frequently than Instagram (*M* = 2.47, *SD* = 2.63). Notably, only 49 participants (4.5%) reported never using Facebook, while 426 participants (38.7%) indicated never using Instagram.

### Measures

#### Dependent Variables: Frequency of Information Use on Social Media

To assess different types of information use on social media, we apply the four information needs proposed by Hasebrink and Domeyer (2010), which have been used to conceptualize different types of information use across media types (e.g., Hölig, 2013) and on social media (Kümpel et al., 2022). Building on these studies, we developed a scale in which participants indicated how frequently they use either Facebook or Instagram to access content from various domains corresponding to the four information needs. Responses were provided on an 8-point scale ranging from *never* (1) to *several times a day* (8). For each of the four information needs, two items were used to measure the frequency of related information use (see below). The prompt given to participants was:

“On [Platform] you come across content from a wide variety of areas. Please indicate how often you approximately use content from the following areas on [Platform]. By ‘use’ we mean actions such as briefly skimming a post, reading it in detail, commenting on it, or sharing it.”

Depending on participants’ self-reported social media use and response rates for this question, one of the two platforms (Facebook or Instagram) was used in place of “[Platform].” For instance, if a participant reported using both Facebook and Instagram, and there was less data for Facebook at that point in the survey, the participant was asked about Facebook. This approach aimed to ensure a relatively equal number of responses for both platforms, which was achieved successfully (*n*_*Facebook*_ = 563, *n*_*Instagram*_ = 537).

##### Frequency of Undirected Information Use on Social Media

The two items corresponding to *undirected* information needs were “News from politics and society (e.g., elections, sporting events)” and “General content related to life management (e.g., legal regulations, behavior in the event of a disaster).” A mean index of both items was calculated for further analyses (*M* = 3.25, *SD* = 2.11, *r*_*SB*_ = 0.85).

##### Frequency of Topic-Related Information Use on Social Media

The two items corresponding to *topic-related* information needs were “Own thematic interests (e.g., history, travel, finance)” and “Own hobbies and leisure activities (e.g., handicrafts, gardening, fitness).” A mean index of both items was calculated for further analyses (*M* = 3.52, *SD* = 2.08, *r*_*SB*_ = 0.83).

##### Frequency of Group-Related Information Use on Social Media

The two items corresponding to *group-related* information needs were “Close social environment (e.g., content from family and friends)” and “Wider social environment (e.g., content from acquaintances and colleagues).” A mean index of both items was calculated for further analyses (*M* = 4.20, *SD* = 2.20, *r*_*SB*_ = 0.90).

##### Frequency of Problem-Related Information Use on Social Media

The two items corresponding to *problem-related* information needs were “Content to solve a specific problem (e.g., repair instructions, tips to fight a cold)” and “Content for particular needs (e.g., recipes, product information).” A mean index of both items was calculated for further analyses (*M* = 2.99, *SD* = 1.90, *r*_*SB*_ = 0.88).

The intercorrelations among the four information use dimensions ranged from r = .57 to r = .71, indicating moderate to strong relationships (see Appendix, Figure A-1). This aligns with the theoretical expectations outlined above, as these dimensions represent interconnected yet distinct constructs within a multidimensional framework (Hasebrink & Domeyer, 2010).

#### Independent Variables

##### Basic Psychological Needs

The three basic psychological needs—autonomy, competence, and relatedness—were assessed using the corresponding subscales developed by Karahanna et al. (2018). Each subscale consisted of three items designed to measure one of the needs. Responses were provided on a 7-point scale ranging from *does not apply at all* (1) to *does fully apply* (7). Participants’ overall needs for autonomy (*M* = 6.46, *SD* = 0.72, ω_h_ = .74), competence (*M* = 5.01, *SD* = 1.23, ω_h_ = .72), and relatedness (*M* = 4.70, *SD* = 1.35, ω_h_ = .87) were then calculated with a mean index of the three respective need-related items.

##### Fear of Missing Out (FOMO)

FOMO was assessed using the ten-item Fear of Missing Out Scale developed by Przybylski and Colleagues (2013b). Responses were provided on a 7-point scale ranging from *does not apply at all* (1) to *does fully apply* (7). Participants’ overall FOMO was then calculated with a mean index of all ten items (*M* = 3.07, *SD* = 1.07, ω_h_ = .80).

##### Openness to Experience

Openness to experience was assessed using the corresponding subscale of the Big Five Inventory-SOEP (BFI-S) developed by Schupp and Gerlitz (2008), which includes three items. Responses were provided on a 7-point scale ranging from *does not apply at all* (1) to *does fully apply* (7). Participants’ overall openness to experience was then calculated with a mean index of all three items (*M* = 4.77, *SD* = 1.33, ω_h_ = .75).

##### Political Interest

Political interest was assessed using the five-item Short Scale Political Interest (SSPI) developed by Otto and Bacherle (2011). Responses were provided on a 7-point scale ranging from *does not apply at all* (1) to *does fully apply* (7). Participants’ overall political interest was then calculated with a mean index of all five items (*M* = 4.84, *SD* = 1.67, ω_h_ = .95).

### Analytical Approach

We conducted four hierarchical multiple regression models to address RQ1 and RQ2, each with a different dependent variable (DV):

• **Model 1 (DV):** Frequency of Undirected Information Use on Social Media
• **Model 2 (DV):** Frequency of Topic-Related Information Use on Social Media
• **Model 3 (DV):** Frequency of Group-Related Information Use on Social Media
• **Model 4 (DV):** Frequency of Problem-Related Information Use on Social Media

The models included three blocks of predictors. The first block controlled for demographic variables (age, gender, education) and the social media platform used in the questionnaire (Facebook or Instagram). The second block included the three basic psychological needs (autonomy, competence, relatedness), while the third block added the relevant personality traits—FOMO, political interest, and openness to experience. This stepwise approach allowed us to assess how each set of predictors contributed to the model’s explanatory power while controlling for other variables. To answer both research questions, we focus on the full model with all predictors included. Details of the stepwise models are presented in the Appendix (Tables A-1 to A-4).

## Results

*RQ1* explored how the three basic psychological needs—autonomy, competence, and relatedness—influence the frequency of older adults’ use of different types of information on social media platforms. The findings from our hierarchical multiple regression models revealed a nuanced pattern, with significant effects only observed for the frequency of group-related and undirected information use (Table 1). The strongest predictor derived from self-determination theory was relatedness, which showed a significant positive effect on group-related information use (*β* = 0.16, *p* < .001), and, slightly less pronounced, on undirected information use (*β* = 0.08, *p* = .025). These results indicate that older adults with a higher need for relatedness tend to use social media more frequently to obtain information about their social environment (group-related information) and to access news or general life management information (undirected information). Conversely, autonomy was found to have a small but significant negative effect on undirected information use (*β* = −0.06, *p* = .036). This suggests that older adults with a stronger need for autonomy may be more skeptical about using social media for news or general life management information. Notably, there was no association between the need for competence and any of the four types of information use.

**Table 1.** Linear Regression Models Predicting Older Adults’ Frequency of Four Types of Information Use.

	Undirected	Topic-related	Group-related	Problem-related
(Intercept)	−0.18	−0.18^**^	−0.23^**^	−0.12
Block 1				
Age	−0.04	**−0.10** ^***^	**−0.07** ^**^	−0.03
Gender: Female^a^	−0.03	0.03	0.02	0.1
Education: Medium^b^	0.09	0.09	−0.005	0.008
Education: High^b^	0.01	**0.14** ^*^	0.06	−0.009
Platform: Facebook^c^	**0.31** ^***^	**0.16** ^**^	**0.39** ^***^	**0.14** ^*^
Block 2				
Autonomy	**−0.06** ^*^	−0.03	0.009	−0.05
Competence	0.04	−0.001	0.001	0.001
Relatedness	**0.08** ^**^	0.06	**0.16** ^***^	0.04
Block 3				
FOMO	**0.19** ^***^	**0.19** ^***^	**0.21** ^***^	**0.26** ^***^
Political interest	**0.27** ^***^	**0.13** ^***^	0.05	**0.09** ^**^
Openness to experience	0.01	**0.15** ^***^	0.01	**0.11** ^***^
*R*^2^	0.183	0.138	0.142	0.125
Adj. *R*^2^	0.175	0.129	0.133	0.116

*Note.* Standardized regression coefficients (significant predictors in **bold**), *n* = 1100.

***p < .001; **p < .01; *p < .05.

^a^reference category: not female.

^b^reference category: low.

^c^reference category: Instagram.

The results are more clear-cut for *RQ2*, which explored how the personality traits FOMO, political interest, and openness to experience influence the frequency of older adults’ use of different types of information on social media platforms. FOMO emerged as the most robust predictor, significantly and positively associated with all four types of information use: undirected (*β* = 0.19, *p* < .001), group-related (*β* = 0.19, *p* < .001), topic-related (*β* = 0.21, *p* < .001), and problem-related (*β* = 0.26; *p* < .001). These findings suggest that older adults with higher FOMO use social media more frequently for *all* types of information. Political interest also emerged as an important predictor of older adults’ social media information use, with positive associations observed for undirected (*β* = 0.27, *p* < .001), topic-related (*β* = 0.13, *p* < .001), and problem-related information use (*β* = 0.09, *p* = .004). However, political interest was not associated with group-related information use. Lastly, openness to experience was positively associated with topic-related (*β* = 0.15, *p* < .001) and problem-related information use (*β* = 0.11, *p* < .001), indicating that older adults who are curious and imaginative are more likely to use social media for information related to their hobbies, interests, or problem-solving.

Overall, the final models incorporating all predictor variables showed moderate to strong explanatory power (adjusted *R*^2^ = 0.12–0.18). A comparison of predictor blocks indicates that personality traits (RQ2) accounted for more variance in social media information use than psychological needs (RQ1). Specifically, SDT-related factors contributed *ΔR*^*2*^ = 0.04–0.05, whereas personality traits led to a greater increase in explanatory power (*ΔR*^*2*^ = 0.07–0.10). An exception emerged for group-related information use, where SDT variables—particularly relatedness—explained more variance than personality traits, underscoring its importance for this type of information use (see Appendix, Table A-5).

Beyond the primary research questions, an interesting pattern emerged regarding platform differences. The platform older adults reported their information use for (Facebook or Instagram) was one of the strongest predictors in all four models. Facebook was more frequently used than Instagram across all types of information use, with substantial effects observed for undirected (*β* = 0.31, *p* < .001), topic-related (*β* = 0.16, *p* = .004), group-related (*β* = 0.39, *p* < .001), and problem-related information use (*β* = 0.14, *p* = .012). While this result is generally coherent with the higher relevance of Facebook compared to Instagram among older adults (in our sample and beyond), it could also suggest that they perceive Facebook as the more suitable platform for information use. This will be discussed in more detail in the following section.

## Discussion

Following the increased integration of social media platforms like Facebook and Instagram into older adults’ daily information practices, this study investigated their use of these platforms for different types of information, such as news and problem-solving content. Using a pre-registered, nationally representative study of German Facebook and Instagram users aged 60 and over (*n* = 1100), we examined how basic psychological needs *(RQ1)* and selected personality traits (*RQ2*) related to information behavior––specifically, FOMO, political interest, and openness to experience––predict their information use.

The findings reveal that both relatedness––the need to feel socially connected––and FOMO emerged as key factors influencing the use of various types of information. While relatedness was particularly relevant for group-related information use, the influence of FOMO appears even stronger, as this trait was positively related to all four types of information use.^1^ The importance of these socially oriented factors highlights the social origin of older adults’ social media use. Even when engaging with information that is not explicitly social, this engagement still seems to be tied to relational and emotional needs. This interpretation is supported by prior research showing that older adults often share travel experiences (e.g., Darley et al., 2017) or cultural events (e.g., Bansal & Choudhary, 2023) to foster a sense of connectedness. Thus, their social media use is not only about accessing information but also about maintaining social bonds, suggesting that multiple information needs are often fulfilled simultaneously.

On a more general level––beyond older adults specifically––, it appears plausible that individuals who are more concerned about their social integration use social media more frequently for various types of information (see also Bloemen & De Coninck, 2020; Wu-Ouyang, 2024). Depending on one’s social environment, maintaining social integration may involve staying informed on different issues—such as news within a politically engaged circle or the latest gadget trends in a tech-savvy group. The consistent relevance of FOMO suggests that this trait may also drive a ‘preventive’ information-seeking behavior, where individuals consume diverse content on social media to avoid feeling excluded.

Autonomy—the need to independently regulate one’s actions and experiences—showed a negative association with older adults’ use of undirected information, such as news. Although the effect size was small, this suggests that older adults with a higher need for autonomy likely prioritize staying informed, as past research on younger adults indicates (e.g., Dubnjakovic, 2018). As a result, such older adults may have already established news habits before the widespread availability of social media, making it unnecessary as an additional information source. Furthermore, the algorithmically and socially curated nature of information on Facebook and Instagram (e.g., Kümpel, 2022) may be perceived as limiting autonomy in information selection. Unlike traditional media, where individuals actively choose what to consume, social media exposes users to content shaped by algorithms and peer activity. This curated exposure may create a sense of reduced control over one’s information flow, potentially discouraging older adults from using social media for news and general life management.

In contrast to autonomy and relatedness, competence––the need to feel effective and capable in one’s activities––did not significantly relate to any type of information use. One possible explanation is that competence may be a more relevant predictor of social media adoption *in general*, as older adults with a higher need for competence might be more motivated to explore new technologies. However, our sample only included older adults who were already social media users. Additionally, competence might not be linked to a specific type of information use, as fulfilling this need can involve various types of information. For instance, it may include news articles (undirected information), lunch recipes (problem-related information), or knitting tutorials (topic-related information), depending on individual interests (see also Zhao et al., 2023).

Despite this nuanced pattern, our findings underscore the relevance of SDT (Ryan & Deci, 2017) in explaining older adults’ social media information use. Specifically, the results highlight the role of need strength as a key factor in digital behaviors, demonstrating that while psychological needs are universal, their intensity varies across individuals, shaping distinct patterns of social media information use. More broadly, just as research has explored different levels of need satisfaction as both predictors and outcomes of (digital) media use (e.g., Lee & Lin, 2016; Lin, 2016), our findings suggest that variations in need strength are equally important for understanding diverse digital behaviors.

Personality traits also played an important role in shaping information use patterns. Similar to FOMO, political interest was positively associated with multiple types of information use, particularly undirected information. This finding aligns with prior research across different age groups demonstrating a positive relationship between political interest and news consumption (Shehata & Strömbäck, 2021). However, political interest also predicted the use of topic- and problem-related information. This association may be attributed to a higher need for cognition among politically interested individuals (Condra, 1992). Additionally, politically interested individuals generally exhibit a “stable motivation for new ideas” (Larsen, 2022, p. 467), which could motivate them to use various types of information. Indeed, political interest also correlated with openness to experience (*r* = 0.27), a personality trait characterized by receptiveness to new experiences and ideas. As expected, openness to experience was positively related to older adults’ use of topic- and problem-related information on social media. In summary, these findings suggest that older adults with more (political) interests tend to use social media for a broader range of information.

In several ways, our findings also indicate that the *platforms’ characteristics* influence how older adults use information on social media. Notably, only autonomy was related to a single type of information use, whereas relatedness and all examined personality traits were associated with at least two. This finding may point to older adults incidentally encountering information on Facebook or Instagram. Due to mechanisms of algorithmic and social curation, social media users often stumble upon information they were not consciously looking for (Ahmadi & Wohn, 2018). For instance, while primarily using Facebook to stay connected with friends and family, older adults may come across a colleague’s shared recipe or a trending news article recommended by the platform’s algorithm. As a result, the time spent on social media engaging with one type of information may increase the likelihood of encountering other types as well.

The influence of platforms was also evident in the regression models. The platform older adults reported their information use for (Facebook or Instagram) was a significant predictor across all four types of information use, with Facebook being used more frequently than Instagram. While this may simply reflect Facebook’s higher relevance among older adults in Germany (Müller, 2024), it could also indicate that older adults view Facebook as more suitable for obtaining information. One possible reason is Facebook’s stronger network-oriented structure compared to Instagram (Bossetta, 2018) which may better support group-related information needs, often overlapping with other types of information use. Unlike Instagram’s one-way following system, Facebook fosters mutual friendships, birthday reminders, and event planning. Additionally, Facebook Groups, frequently used by older adults (e.g., Bernardo et al., 2022), provide spaces for connecting with local communities or individuals with shared interests. Finally, previous research suggests that older adults often perceive social media as confusing or chaotic (e.g., Meng, 2024) and that they are particularly overwhelmed by a high density of visual elements (Elguera Paez & Zapata Del Río, 2019)—an issue that may be more pronounced on Instagram. Therefore, it will be crucial to further explore older adults’ perceptions of *different* social media platforms and their unique characteristics, rather than treating social media as a homogeneous analytical category.

Of course, this study has certain limitations. First, its cross-sectional design does not allow for testing causal relationships. While theoretical considerations suggest specific directional relationships, it remains possible that some of the associations reported here are reciprocal. Additionally, this study offers only an initial systematic perspective on how older adults use social media for different types of information. Future research should therefore delve deeper into platform-specific information use to provide a more nuanced understanding.

Similar to news audience research focused on young adults (e.g., Anter & Kümpel, 2023; Swart, 2021), studies should examine the roles of different social media platforms within older adults’ information repertoires in greater detail. This includes investigating how older adults perceive key platform features and how these perceptions relate to their evaluation and use of content. It also involves identifying older adults’ practices and habits of engaging with social media content, such as information “snacking” or continuous “monitoring” (Costera Meijer & Groot Kormelink, 2015, p. 664; see also Molyneux, 2018), and how these behaviors vary according to personality traits. Future research should also include additional factors known to influence (social) media use, such as news media trust (e.g., Kalogeropoulos et al., 2019) or extraversion (e.g., Gil de Zúñiga et al., 2017).

To gain such insights, future studies could employ stimulus-based qualitative designs, such as using think-aloud protocols (Freiling, 2019). Finally, this study did not investigate the specific content older adults engage with on social media. Addressing this gap could involve inviting participants to donate existing digital trace data, which can offer valuable insights into algorithmic curation and exposure to specific types of information (e.g., Thorson et al., 2021).

## Supplemental Material

Supplemental Material - Older Adults’ Information Use on Social Media: The Role of Psychological Needs and Personality Traits Supplemental Material for Older Adults’ Information Use on Social Media: The Role of Psychological Needs and Personality Traits by Luise Anter, Martin Fischer and Anna Sophie Kümpel in Research on Aging.

## Data Availability

Data, materials, and scripts to reproduce the analyses are available at https://doi.org/10.17605/OSF.IO/SD2T5 (Anter et al., 2024)

## Note

1. However, it has to be noted that relatedness and FOMO are correlated (*r* = .40; see Figure A-2 in the Appendix), which is why each predictor’s effect on the dependent variables will include some shared variance with other correlated predictors.
