# Supplementary material for: Older Adults’ Information Use on Social Media: The Role of Psychological Needs and Personality Traits
Source: Res Aging. 2025 May 14;47(9-10):471–81. doi: 10.1177/01640275251341447 (PMC12391613; doi:10.1177/01640275251341447)
Supplement: Supplemental Material - Older Adults’ Information Use on Social Media: The Role of Psychological Needs and Personality Traits [file sj-pdf-1-roa-10.1177_01640275251341447.pdf]

Older Adults' Information Use on Social Media: The Role of Psychological Needs and Personality Traits - Appendix

Figure A-1

Correlation Matrix for Dependent Variables

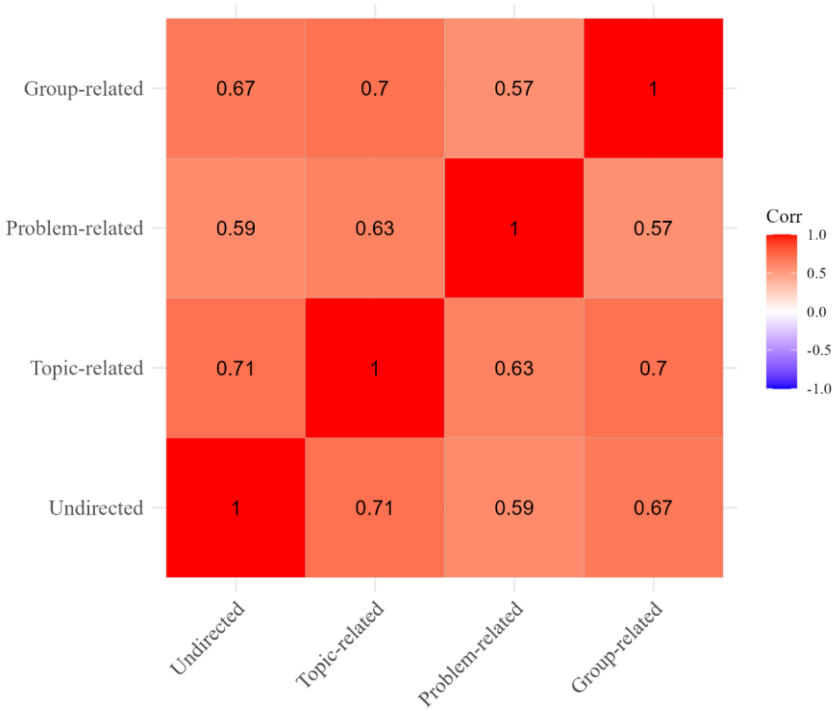

Figure A-2

Correlation Matrix for Predictor Variables

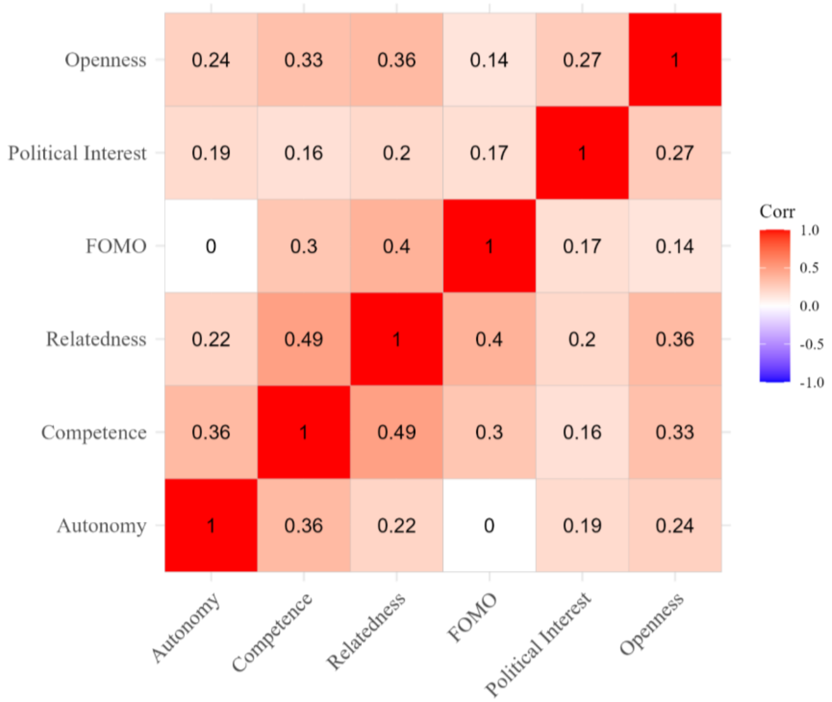

Table A-1

Linear Regression Models Predicting Older Adults' Frequency of Using Undirected Information

		**Undirected Information**		
		**Controls**	**SDT**	**Personality**
	(Intercept)	**-0.17** (0.07)*	**-0.16** (0.07)*	**-0.18** (0.06)**
Block 1				
	Age	-0.01 (0.03)	-0.02 (0.03)	-0.04 (0.03)
	Gender: Female[a]	**-0.13** (0.06)*	**-0.17** (0.06)**	-0.03 (0.06)
	Education: Medium[b]	**0.18** (0.07)*	**0.17** (0.07)*	0.09 (0.07)
	Education: High[b]	**0.14** (0.07)*	0.13 (0.07)	0.01 (0.07)
	Platform: Facebook[c]	**0.26** (0.06)***	**0.27** (0.06)***	**0.31** (0.06)***
Block 2				
	Autonomy		-0.05 (0.03)	**-0.06** (0.03)*
	Competence		**0.09** (0.04)**	0.04 (0.03)
	Relatedness		**0.19** (0.03)***	**0.08** (0.03)*
Block 3				
	FOMO			**0.19** (0.03)***
	Political Interest			**0.27** (0.03)***
	Openness to Experience			0.01 (0.03)
R^2		0.02	0.08	0.18
Adj. R^2		0.02	0.07	0.17

Note: Standardized regression coefficients (significant predictors in **bold**, standard deviation in brackets), $n = 1{,}100$; [a] reference category: not female; [b] reference category: low; [c] reference category: Instagram; ***$p < 0.001$; **$p < 0.01$; *$p < 0.05$.

Table A-2

Linear Regression Models Predicting Older Adults' Frequency of Using Topic-Related Information

		Topic-Related Information		
		**Controls**	**SDT**	**Personality**
	(Intercept)	**-0.20** (0.07)**	**-0.18** (0.07)**	**-0.18** (0.06)**
Block 1				
	Age	**-0.09** (0.03)**	**-0.10** (0.03)***	**-0.10** (0.03)***
	Gender: Female[a]	0.01 (0.06)	-0.03 (0.06)	0.03 (0.06)
	Education: Medium[b]	**0.15** (0.07)*	**0.15** (0.07)*	0.09 (0.07)
	Education: High[b]	**0.24** (0.07)**	**0.23** (0.07)**	**0.14** (0.07)*
	Platform: Facebook[c]	0.11 (0.06)	**0.12** (0.06)*	**0.16** (0.06)**
Block 2				
	Autonomy		-0.01 (0.03)	-0.03 (0.03)
	Competence		0.06 (0.04)	-0.00 (0.03)
	Relatedness		**0.18** (0.03)***	0.06 (0.04)
Block 3				
	FOMO			**0.19** (0.03)***
	Political Interest			**0.13** (0.03)***
	Openness to Experience			**0.15** (0.03)***
R^2		0.02	0.07	0.14
Adj. R^2		0.02	0.06	0.13

Note: Standardized regression coefficients (significant predictors in **bold**, standard deviation in brackets), $n = 1{,}100$; [a] reference category: not female; [b] reference category: low; [c] reference category: Instagram; ***$p < 0.001$; **$p < 0.01$; *$p < 0.05$.

Table A-3

Linear Regression Models Predicting Older Adults' Frequency of Using Group-Related Information

		Group-Related Information		
		**Controls**	**SDT**	**Personality**
	(Intercept)	**-0.21** (0.07)**	**-0.19** (0.07)**	**-0.23** (0.06)***
Block 1				
	Age	-0.05 (0.03)	**-0.07** (0.03)*	**-0.07** (0.03)*
	Gender: Female[a]	0.01 (0.06)	-0.03 (0.06)	0.02 (0.06)
	Education: Medium[b]	0.01 (0.07)	0.01 (0.07)	-0.01 (0.07)
	Education: High[b]	0.07 (0.07)	0.06 (0.07)	0.06 (0.07)
	Platform: Facebook[c]	**0.35** (0.06)***	**0.36** (0.06)***	**0.39** (0.06)***
Block 2				
	Autonomy		-0.01 (0.03)	0.01 (0.03)
	Competence		0.05 (0.03)	-0.00 (0.03)
	Relatedness		**0.24** (0.03)***	**0.16** (0.03)***
Block 3				
	FOMO			**0.21** (0.03)***
	Political Interest			0.05 (0.03)
	Openness to Experience			0.01 (0.03)
R^2		0.03	0.10	0.14
Adj. R^2		0.03	0.09	0.13

Note: Standardized regression coefficients (significant predictors in **bold**, standard deviation in brackets), n = 1,100; [a] reference category: not female; [b] reference category: low; [c] reference category: Instagram; ***$p < 0.001$; **$p < 0.01$; *$p < 0.05$.

Table A-4

Linear Regression Models Predicting Older Adults' Frequency of Using Problem-Related Information

		Problem-Related Information		
		**Controls**	**SDT**	**Personality**
	(Intercept)	-0.11 (0.07)	-0.10 (0.07)	-0.12 (0.06)
Block 1				
	Age	-0.02 (0.03)	-0.03 (0.03)	-0.03 (0.03)
	Gender: Female[a]	0.06 (0.06)	0.03 (0.06)	0.10 (0.06)
	Education: Medium[b]	0.06 (0.08)	0.05 (0.07)	0.01 (0.07)
	Education: High[b]	0.04 (0.07)	0.03 (0.07)	-0.01 (0.07)
	Platform: Facebook[c]	0.09 (0.06)	0.10 (0.06)	**0.14** (0.06)*
Block 2				
	Autonomy		-0.05 (0.03)	-0.05 (0.03)
	Competence		**0.07** (0.04)*	0.00 (0.04)
	Relatedness		**0.17** (0.03)***	0.04 (0.04)
Block 3				
	FOMO			**0.26** (0.03)***
	Political Interest			**0.09** (0.03)**
	Openness to Experience			**0.11** (0.03)***
R^2		0.00	0.05	0.13
Adj. R^2		-0.00	0.04	0.12

Note: Standardized regression coefficients (significant predictors in **bold**, standard deviation in brackets), $n = 1{,}100$; [a] reference category: not female; [b] reference category: low; [c] reference category: Instagram; ***$p < 0.001$; **$p < 0.01$; *$p < 0.05$.

Table A-5

Comparison of All Four Hierarchical Models with Explained Variance

Dependent Variable	Block	Adj. R^2	ΔR^2
Frequency of Using **Undirected** Information	Controls	0.02	
	SDT	0.07	0.05
	Personality	0.18	0.10
Frequency of Using **Topic-related** Information	Controls	0.02	
	SDT	0.06	0.04
	Personality	0.13	0.07
Frequency of Using **Group-related** Information	Controls	0.03	
	SDT	0.09	0.07
	Personality	0.13	0.04
Frequency of Using **Problem-related** Information	Controls	0.00	
	SDT	0.04	0.04
	Personality	0.12	0.08
